# Biochemical Characterization of Glutamate Racemase—A New Candidate Drug Target against *Burkholderia cenocepacia* Infections

**DOI:** 10.1371/journal.pone.0167350

**Published:** 2016-11-29

**Authors:** Aygun Israyilova, Silvia Buroni, Federico Forneris, Viola Camilla Scoffone, Namiq Q. Shixaliyev, Giovanna Riccardi, Laurent Roberto Chiarelli

**Affiliations:** 1 Dipartimento di Biologia e Biotecnologie, Università degli Studi di Pavia, Pavia, Italy; 2 Department of Microbiology, Baku State University, Baku, Azerbaijan; 3 Department of Organic Chemistry, Baku State University, Baku, Azerbaijan; Weizmann Institute of Science, ISRAEL

## Abstract

The greatest obstacle for the treatment of cystic fibrosis patients infected with the *Burkholderia* species is their intrinsic antibiotic resistance. For this reason, there is a need to develop new effective compounds. Glutamate racemase, an essential enzyme for the biosynthesis of the bacterial cell wall, is an excellent candidate target for the design of new antibacterial drugs. To this aim, we recombinantly produced and characterized glutamate racemase from *Burkholderia cenocepacia* J2315. From the screening of an in-house library of compounds, two Zn (II) and Mn (III) 1,3,5-triazapentadienate complexes were found to efficiently inhibit the glutamate racemase activity with IC_50_ values of 35.3 and 10.0 μM, respectively. Using multiple biochemical approaches, the metal complexes have been shown to affect the enzyme activity by binding to the enzyme-substrate complex and promoting the formation of an inhibited dimeric form of the enzyme. Our results corroborate the value of glutamate racemase as a good target for the development of novel inhibitors against *Burkholderia*.

## Introduction

Cystic fibrosis (CF) is a rare inherited disease, caused by the malfunction of the chloride channel Cystic Fibrosis Transmembrane conductance Regulator (CFTR). Persistent lung infections are responsible for irreversible bronchiectasis and respiratory failure, and represent the main cause of morbidity and mortality in CF individuals [1–4]. One group of bacteria that causes life-threatening infections in CF people belongs to the *Burkholderia cepacia* complex (Bcc), and is responsible for the “Cepacia Syndrome” which leads to a rapid deterioration of lung function and affects the life expectancy of CF patients [5]. The treatment of patients with Bcc is particularly difficult because of flexible genome structure and diverse metabolic activity: bacteria can produce a wide variety of potential virulence factors and exhibit innate or acquired resistance to many commonly used antibiotics and disinfectants. The complex is resistant to a wide range of antibiotic classes including polymyxins, aminoglycosides, trimethoprim, quinolones and β-lactams, as well as antimicrobial peptides of the host [3, 6, 7]. Several resistance mechanisms have been reported in *B*. *cenocepacia*, such as enzymatic inactivation, modification of drug target, cell wall permeability, various efflux pumps and biofilm formation [6, 8–11]. Consequently, nowadays the discovery of new compounds able to inhibit the growth of *B*. *cenocepacia*, as well as of new potential drug targets, is considered one of the most prominent problems.

In this context, glutamate racemase (GR, EC 5.1.1.3), an essential enzyme which is absent in humans, is a good candidate target. This cofactor-independent enzyme belongs to the two-thiol based family of Asp/Glu amino acid racemases and catalyzes the interconversion of L-glutamate (L-Glu) to D-glutamate (D-Glu) [12, 13], an essential component of the peptidoglycan layer of the cell wall of several pathogens.

Peptidoglycan biosynthesis is a complex process, which can be divided into three phases with different cellular localization. The pathway starts with the synthesis of the nucleotide precursors (phase I) which occurs in the cytoplasm. The second phase, the synthesis of the lipid-linked intermediates, is performed on the inner side of the cytoplasmic membrane, while the third phase (polymerization reactions) at the outer side of the cytoplasmic membrane [14, 15].

Most active drugs inhibit the phase III pathway, which involves the extracellular cross-linking and maturation of the cell envelope. However, due to the raise of bacterial resistance to compounds targeting phase III, new active compounds against *Burkholderia*, preferably with unprecedented modes of action, are needed. In this respect, targeting enzymes like GR, involved in phase I, could represent a new promising strategy [16].

Structural and functional studies of GR from different bacteria, including *Helicobacter pylori* [17], *Bacillus subtilis* [18], *Streptococcus pyogenes* [19], *Lactobacillus brevis* [20], *Aquifex pyrophilus* [12] and *Bacillus anthracis* [21], revealed a high divergence among glutamate racemase enzymes. In fact, several different drugs have been reported as GR inhibitors, such as pyrazolopyrimidinediones [13], pyridodiazepine amines [17], 8-benzyl pteridine-6,7-diones [22], dipicolinate and benzoat-3-sulfonate [23], (2R,4S)-4-substituted D-glutamate analogs [18], 1-H-benzimidazole-2-sulfonic acid [24], 2,6 pyridinedicarboxylic acid [23, 25] and 4-hydroxybenzene-1,3-disulfonate [26].

In this study, we focused on GR from *B*. *cenocepacia* J2315 (*Bc*GR), and we performed library screening for the identification of compounds targeting the recombinant enzyme *in vitro*. We report on the identification of two Zn (II) and Mn (III) 1,3,5-triazapentadienate complexes [27, 28] that inhibit *Bc*GR, showing an unusual mechanism of action.

## Materials and Methods

### Chemicals

DNase I, NAD^+^, *p*-iodonitrotetrazolium violet (INT), L- and D-glutamic acid, L-glutamate dehydrogenase, diaphorase, isopropyl-β-D thiogalactopyranoside (IPTG), bovine serum albumin (BSA), chicken egg lysozyme, kanamycin were purchased from Sigma-Aldrich. Other chemicals were reagent grade.

### Bacterial strains, media, growth condition, and plasmids

Cloning steps were performed in *Escherichia coli* Stellar^TM^ competent cells according to the protocol of the In-Fusion HD Cloning kit (Takara). *E*. *coli* BL21 (DE3) and pET-28a(+) expression plasmid (Novagen) were used for overproduction of recombinant protein. Cells were grown in Luria-Bertani (LB) medium at 37°C with shaking (200 rpm) in the presence of antibiotic (kanamycin 50 μg/ml).

### Cloning, expression, and purification of *Burkholderia cenocepacia* glutamate racemase

*BCAL2289* gene, encoding the *B*. *cenocepacia* J2315 glutamate racemase (*Bc*GR) was amplified by PCR, using the following primers: 2289_for (5’- ATGGGTCGCGGATCCCTGGAAGTTCTGTTCCAGGGGCCCATGACGAACCCGTCCGAC—3’) and 2289_rev (5’- CTCGAATTCGGATCCTCAGGCGGTCGCGCAGGC—3’). The primers were designed according to the In-fusion HD Cloning Kit protocol; the forward primer carried PreScission Protease cleavage site (underlined) in order to remove the 6-histidine tag from the protein. The purified PCR fragment was recombined into the *Hind*III-*Eco*RI digested pET-28a(+) following the manufacturer’s instructions, to give pET28a_*BcGR* vector. The recombinant products were transformed into *E*. *coli* Stellar^TM^ competent cells and the resulting colonies were checked for the presence of insert by colony PCR and sequencing.

For protein expression, *E*. *coli* BL21(DE3) strain was transformed with pET-28a_ *BcGR*, plated onto LB-kanamycin (50 μg/ml) and a single colony was used as a starter culture. One liter of LB-kanamycin was inoculated with 20 ml of starter culture and grown at 37°C until OD_600_ = 0.6. Protein expression was induced with 0.5 mM IPTG at 25°C for 15 h.

Cells were harvested by centrifugation, resuspended in 50 mM Tris-HCl pH 8.0, 100 mM NaCl, 2 mM dithiothreitol (DTT) (Buffer A) and lysed by sonication. The cell-free extract, obtained by centrifuging the lysate at 30000 *g* for 45 minutes at 4°C, was loaded on nickel nitrilotriacetic acid resin (Ni-NTA, Qiagen) equilibrated in buffer A and packed in a column, the column washed with 20 mM imidazole in buffer A, and *BcGR* eluted with 50–100 mM imidazole.

The purified enzyme was dialyzed in 50 mM Tris-HCl pH 8.0, 100 mM NaCl, 2 mM DTT, and digested with PreScission protease (GE Healthcare, 400 mU/ml). The digested protein was further purified by a second affinity chromatography, in the same buffer. Samples purity was checked by SDS-PAGE and protein concentration evaluated by absorbance at 280 nm (ε = 40715 M^-1^ cm^-1^).

### Analytical gel filtration analysis

The relative molecular mass of native *Bc*GR was determined in the presence and absence of ligands: L-Glu (10 mM), D-Glu (10 mM). To this purpose repeated injections (50 μl) of protein (0.5 mg ml^-1^) were applied through an autosampler (Shimadzu) onto a Superdex 75 5/150 GL column (GE Healthcare), equilibrated in 50 mM Tris-HCl pH 8.0, 150 mM KCl, 2mM DTT, at a flow rate of 0.1 ml min^-1^, using a Prominence automated HPLC system coupled to UV-Vis absorbance and fluorescence detectors (Shimadzu). All runs were performed at 16°C constant temperature. Protein elution was monitored by following absorbance at 280 nm and tryptophan fluorescence (excitation 280 nm, emission 335 nm). Column calibration was performed with the following internal standards: *B*. *cenocepacia* CepI (22 kDa), *M*. *tuberculosis* Rv2466c (46 kDa), *M*. *tuberculosis* pantothenate kinase (71 kDa), and *M*. *tuberculosis* CTP synthetase (254 kDa).

### Enzymatic activity assays, steady state kinetics and inhibition assays

Enzymatic activity of *Bc*GR was determined in the reverse direction using D-Glu as a substrate, by a spectrophotometric coupled assay in the presence of L-glutamate dehydrogenase and diaphorase [29]. The assays were performed with an Eppendorf Biospectrometer, in a final volume of 100 μl at 37°C. The standard reaction mixture (100 μl) contained 50 mM HEPES pH 8.0, 5 mM NAD^+^, 37.5 units of bovine L-glutamate dehydrogenase, 2.5 mM ADP, 0.65 mM INT, 2 units of diaphorase, 20 mM D-Glu, and the reactions were started by the addition of *Bc*GR enzyme (18 μM final concentration). One unit is defined as the amount of enzyme catalyzing the conversion of 1 μmol of D-Glu per min, in the above conditions.

Steady-state kinetic parameters were determined by assaying the enzyme at variable concentrations of D-Glu (5–50 mM). The experiments were performed in triplicate, and the kinetic constants, *K*_m_, and *k*_cat_ were determined by fitting the data to the Michaelis-Menten equation using Origin 8 software.

Initially, *Bc*GR inhibition was screened for all compounds at 100 μM (dissolved in DMSO), at 20 mM D-Glu. IC_50_ was determined for compounds that significantly inhibited the enzyme activity. To this purpose, the enzyme activity was measured in the presence of different concentrations of the compound and estimated according to the Eq 1, where A_[I]_ is the enzyme activity at inhibitor concentration [I] and A_[0]_ is the enzyme activity without inhibitor [30].

A[I]=A[0]x(1−[I]/([I]+IC50)) (1)

To determine the mode of inhibition of the compound, steady state kinetic analysis of *Bc*GR was performed in the presence of different concentrations of the compounds (5–100 μM).

### Thermal stability assays

The influence of temperature on the activity of *Bc*GR was investigated by measuring the enzymatic activity as described above, varying the temperatures from 20°C to 55°C. The effect of pH on the enzyme activity was examined with different buffers in the pH range of 6.0–10.0.

Thermal stability was measured by incubating the enzyme (0.5 mg ml^-1^) at given temperatures in 50 mM HEPES pH 8.0 for up to 24 h. The pH stability was screened by incubating *Bc*GR at 4°C for up to 24 h at different pH (6.0–10.0). In both cases, samples were removed at intervals and residual enzyme activity determined.

### Homology modeling and structural bioinformatics

The sequence of *Bc*GR was downloaded from the UniProt server [31] and used for protein BLAST using the Protein Data Bank as reference database [32]. Homologous racemases with percentages of sequence identity higher than 29% are listed in S1 Table. The *Bc*GR homology model was generated using the modeling tools in HHPRED [33] and MODELLER [34], based on the structures of *M*. *smegmatis* MurI (PDB ID 5JWV, 40% identity), *E*. *coli* MurI (PDB ID 2JFN, 38% identity), and *B*. *anthracis* RacEI (PDB ID 2DWU, 32% identity). Final model quality was assessed using PDBSUM [35] and the Qmean server [36]. Electrostatic potentials were calculated using APBS [37]. Structural figures were generated with PyMol [38].

### Determination of the effect of the compounds on the *Bc*GR oligomeric state

The influence of Zn (II) and Mn (III) 1,3,5-triazapentadienate complexes on the oligomeric state of *Bc*GR in the presence of substrates (D-, L-glutamate) was determined by size exclusion chromatography, chemical cross-linking and blue native (BN) gel electrophoresis.

Size exclusion chromatography was firstly performed as in “Analytical gel filtration” section, after incubating *Bc*GR (0.033 mM) with 0.1 mM compound **(1)** or **(2)**, in the absence or in the presence of D-Glu (10 mM). Additionally, EDTA (10 mM) was added to all samples, to avoid effects of free ions (Zn^2+^ and Mn^3+^) in the metal complexes. To check for the possibility of unspecific cross-linking effects of the compounds, BSA and lysozyme were used as control proteins.

Furthermore, in order to extensively examine the results obtained from HPLC system, samples were also run on a Superdex 75 10/300 GL (GE Healthcare) column, equilibrated with sodium phosphate buffer (50 mM, pH 8.0) containing KCl (150 mM) and DTT (1 mM). In this case protein samples (0.084 mM, in a final volume of 500 μl) were incubated with metal complexes in 1:3, 1:4 and 1:5 molar ratio, in the presence or in the absence of 30 mM D-Glu. After incubation, the samples were loaded on the gel filtration column using Äkta FPLC system (GE healthcare). Protein elution was performed at a flow rate of 0.5 ml min^-1^ and followed by measuring the absorbance at 280 nm; fractions of 0.4 ml were collected. The fractions containing protein were then analyzed by chemical cross-linking and BN gel electrophoresis.

Chemical cross-linking was performed with ethylene glycol disuccinate di (N-succinimidyl) ester (EGS), according to [39]. EGS, dissolved in DMF, was added to the enzyme samples at a final concentration of 1 mM in a total volume of 25 μl. The reaction was incubated for 60 min in sodium phosphate buffer (50 mM, pH 8.0) at room temperature and quenched by adding the SDS-PAGE loading buffer [40]. The efficiency of cross-linking was evaluated by SDS-PAGE (10%) analysis.

Discontinuous native-PAGE was performed as described by Whalen and co-workers with minor modifications [25]. Protein samples were incubated in loading buffer (100 mM Tris-Cl, 40% glycerol, 0.5% Coomassie Brilliant Blue G, pH 8.0) at room temperature for 30 min and applied to 10% BN-polyacrylamide gels (30:0.8 total acrylamide:bis-acrylamide ratio) containing 250 mM Tris-Cl (pH 8.8). Gels were run at constant voltage (100 V) for about 4.5 h at 4°C. The gels were destained with several changes of 10% methanol, 6% acetic acid solution.

### Determination of Minimal Inhibitory Concentrations

The Minimal Inhibitory Concentrations (MIC) of the Zn (II) and Mn (III) 1,3,5-triazapentadienate complexes were assessed against *B*. *cenocepacia* J2315 by using the 2-fold microdilution method in U-bottom 96-well microtiter plates [41].

Briefly, about 10^5^ colony forming units (CFU) were used to inoculate each well of the microplate containing concentrations of compounds ranging from 8 to 1024 μg/ml. Growth was determined by the resazurin method after two days of incubation at 37°C. 30 μl of a solution of resazurin sodium salt (Sigma Aldrich) at 0.01% in distilled water were added to each well, and the microtiters were reincubated at 37°C for about 4 h. The MIC value was defined as the lowest concentration of the compound that prevented a color change from blue to pink.

## Results

### Main features of *Bc*GR

The recombinant enzyme was expressed in *E*. *coli* BL21(DE3) cells, and purified to homogeneity as described in “Materials and Methods”. The typical yield was about 8 mg of purified protein from 8–10 grams of wet cell pellet, with a specific activity of 2.4 U/mg.

**Fig 1:**
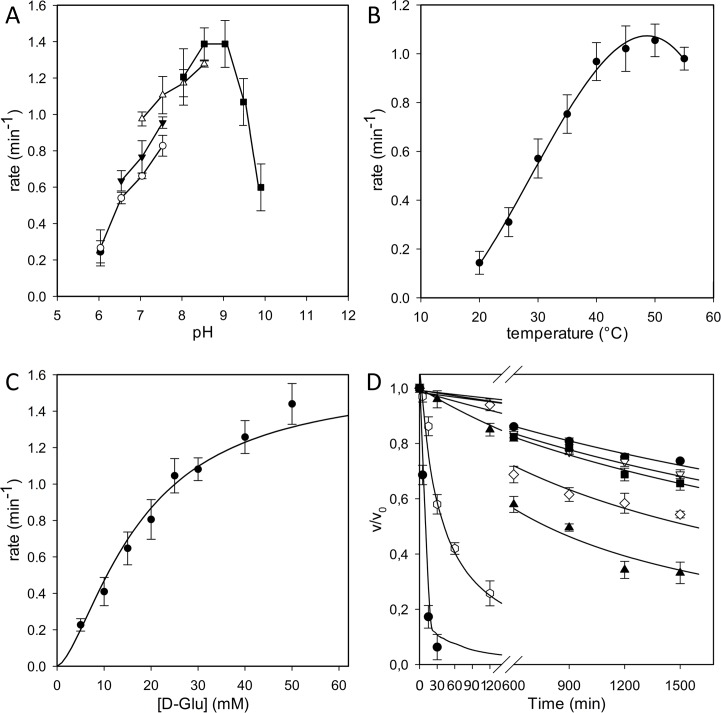
Enzymatic characterization of *Bc*GR. (A) Effect of pH on the activity of *Bc*GR. All measurements were performed as in Materials and Methods, but using the following buffers at 50 mM: MES (pH range 6.0–7.0, ●); sodium phosphate (6.0–8.0, ○); HEPES (7.0–8.5, ▼); Tris-HCl (7.5–9.0, Δ) and glycylglycine (8.5–10.0). (B) Effect of temperature on the activity of *Bc*GR. The measurements were performed at temperatures ranging from 20°C to 55°C. (C) Steady-state kinetics using D-Glu as a substrate. All the activity measurements are the average of at least three independent determinations. (D) Thermal stability determination. The enzyme was incubated at 20 (●), 25 (○), 30 (■), 35 (□), 40 (▲), 45 (△) and 50 (◆) °C. Samples were removed at intervals, chilled on ice and spectrophotometrically assayed, as reported in Materials and Methods.

The pH-activity profile for *Bc*GR (Fig 1A) indicates that the enzyme exhibits a preference for high pH values, showing an optimal activity at pH comprised between 8.0 and 9.0, with about 70% of its maximal activity at pH 9.5, and less than 50% below pH 7.0. Moreover, the enzyme had the higher activity in a range of temperature between 40°C and 50°C, and showing about 80% of its maximal activity at 37°C (Fig 1B).

Steady state kinetic analysis, performed for the D-Glu → L-Glu reaction in HEPES buffer pH 8.0 at 37°C, showed a *k*_cat_ value of 1.5 ± 0.3 min^-1^ and a *K*_m_ value for D-Glu of 13.89 ± 0.61 mM (Fig 1C).

The enzyme stability was firstly evaluated at different pH, by incubating the enzyme at 4°C for up to 24 h in buffers at pH ranging from 6.0 to 10.0, and determining the residual activity. In these conditions, full activity was almost preserved at pH values comprised between 7.0 and 8.5, whereas the stability dramatically decreased at pH values below 6.0 or up to 9.5, with less than 40% of the initial activity left (data not shown). Thus, the thermal stability was evaluated at pH 8.0, revealing that *Bc*GR is a moderately stable enzyme since it preserved more than 80% of the initial activity when incubated for two hours up to 40°C, but it rapidly lost all the activity at higher temperatures (Fig 1D).

**Fig 2:**
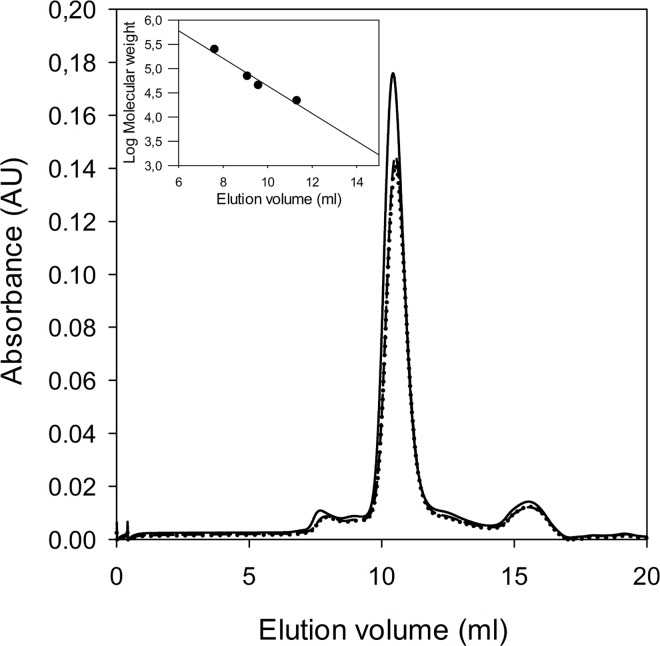
Analytical gel filtration chromatography. Analytical gel filtration chromatography of *Bc*GR was performed in the absence of substrates (solid line) and in the presence of 10 mM D-Glu (dashed line) or L-glutamate (dotted line). The inset represents the molecular weight calibration curve, performed as in Materials and Methods.

The quaternary structure of glutamate racemases is different in various bacteria, being monomeric [13], dimeric [19, 42], or in an equilibrium between these two states [42, 43]. Previous reports indicate that GR oligomerization critically depends on electrostatic interactions on the enzyme surface, and also on the absence or presence of substrate. Notably, numerous different dimerization interfaces were proposed, involving so-called head-to-head or tail-to-tail interactions [12, 18, 19, 21, 42–44]. Despite numerous crystallization attempts, we could not obtain experimental structural information of *Bc*GR either in absence nor in presence of the inhibitors. Thus, we took advantage of the highly conserved molecular architecture of multiple GR structures, to generate a homology model of *Bc*GR for comparative structural analysis (S1 Fig). In *Bc*GR, all critical residues for enzymatic activity and substrate recognition are fully conserved within the 2-domain GR fold (S1 Fig). Comparison of the GR surface charge distribution with the *Bc*GR homology shows significant that differences in charge distribution with both reported head-to-head and tail-to-tail dimeric arrangement (S2 Fig), suggesting that dimerization might not play a functional role in this enzyme. To prove this hypothesis, we experimentally determined the native oligomerization state of *Bc*GR both in presence or absence of 10 mM D,L-Glu by size-exclusion chromatography on Superdex 75 5/150 GL column, as in Materials and Methods. The enzyme was found in monomeric form in all conditions tested, eluting as a sharp peak at a volume corresponding to a molecular weight of about 31 kDa (Fig 2).

### Glutamate racemase inhibition assays

To explore the drugability of the *Bc*GR enzyme, a series of compounds (1H-benzimidazole-2-sulfonic acid, dipicolinic acid, 4-hydroxybenzene-1,3-disulfonate, and (2R)-2-amino-4-benzylpentanedioic acid) [24, 45, 46] which are already known inhibitors of different bacterial glutamate racemases, were screened for the inhibition of the enzymatic activity. Among all compounds tested, only dipicolinic acid was found to be a very weak inhibitor of *Bc*GR, showing an IC_50_ value of 0.15 ± 0.03 mM (S3 Fig).

**Fig 3:**
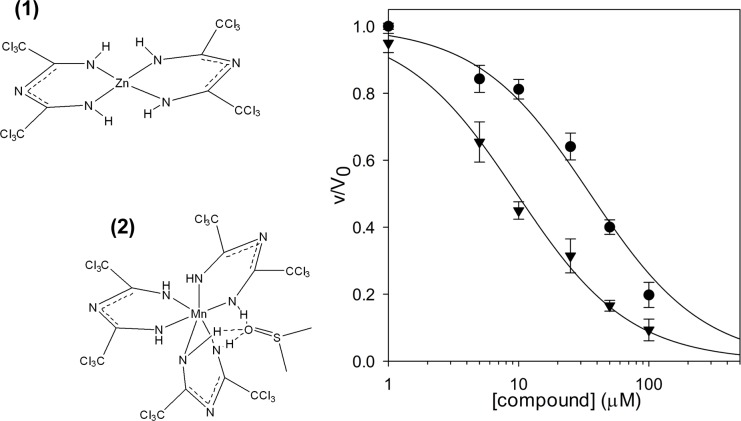
Inhibition of *Bc*GR activity by compounds (1) and (2). IC_50_ value for bis(2,4-bis(trichloromethyl)-1,3,5-triazapentadienate)-zinc(II) complex **(1)** (●), and for tris(2,4-bis(trichloromethyl)-1,3,5-triazapentadienate)-Mn(III) complex **(2)** (▼) was determined at 20 mM of D-Glu, by fitting the experimental data as reported in Materials and Methods.

Afterwards, we screened an in house chemical library. This library is composed of 195 compounds belonging to various chemical classes. The compounds were selected from different whole cells screening and showed diverse antimicrobial activities; for most of these molecules the cellular target is still unidentified. In particular, the collection contains mainly antimycobacterial compounds such as thienopyrimidines [47], quinoxalines [48] and thiophenecarboxamide [49] derivatives, and compounds such as the halogen-1,3,5-triazapentadienate complexes [27, 28, 50], and the thiopyridines [51] and benzothiadiazol [52] analogues, with demonstrated activity also against different Gram positive and negative bacteria. All compounds were tested at a final concentration of 100 μM and among them two, the bis(2,4-bis(trichloromethyl)-1,3,5-triazapentadienato)-zinc(II) complex **(1)** and the tris(2,4-bis(trichloromethyl)-1,3,5-triazapentadienate)-Mn(III) complex **(2)** (Fig 3), were found to almost completely inhibit *Bc*GR.

The IC_50_ values were determined and resulted to be 35.3 ± 4.9 μM and 10.0 ± 0.8 μM for **(1)** and **(2)**, respectively (Fig 3). Moreover, the kinetic analysis of the enzyme in the presence of different concentrations of compounds revealed that they behave as non-competitive inhibitors, causing a decrease in the *V*_max_ values, but leaving the affinity of the enzyme for the substrate unchanged (S4 Fig).

As both compounds carried central metal ions, to avoid any possible effects of free ions on the enzyme activity the inhibition was assayed in the presence of 100-fold excess of ethylenediamine tetra acetic acid (EDTA). The IC_50_ values were only increased of about two folds, confirming that the observed inhibition is more related to ligands rather than to possible free ions released in the solution.

Nonetheless, the effect of different divalent ions (Mg^2+^, Mn^2+^, Fe^2+^, Ni^2+^, and Zn^2+^) on the activity of *Bc*GR was also evaluated, by assaying the enzyme in the presence of their chloride salts. Among them, three (Mn^2+^, Cu^2+^ and Zn^2+^) were able to affect the enzymatic activity (S5 Fig), and in all cases the inhibition was completely abolished in the presence of EDTA, thus confirming a direct role for divalent ions. However, no effects were detected in the presence of the other ions.

### 1,3,5-triazapentadienate metal complexes modulate the oligomeric state of *Bc*GR

To further understand the inhibition mechanism of *Bc*GR by metal complexes, we investigated the oligomeric state of the protein in presence of these compounds by using gel filtration chromatography, upon incubation with **(1)** or **(2)**, with and without addition of D-Glu.

**Fig 4:**
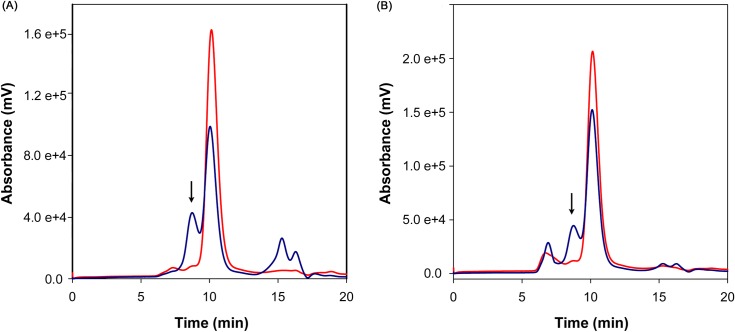
Effect of compound (1) or (2) on the oligomeric state of *Bc*GR. *Bc*GR (33 μM) was incubated with 100 μM of compound **(1)** (Panel A) or **(2)** (Panel B) in the absence (red line) or in the presence of 10 mM D-Glu (blue line), and loaded on a Superdex 75 5/150 GL column, as in Materials and Methods. The peak corresponding to dimeric species (~70 kDa) is indicated with an arrow in both chromatograms. Integration of the peaks revealed a 2:1 monomer-dimer ratio for compound **(1)** (peak areas 1.6 x 10^4^ mV^2^ and 4.4 x 10^3^ mV^2^, for the monomer and dimer area, respectively), and 3:1 for compound **(2)** (peak areas 1.0 x 10^4^ mV^2^ and 4.2 x 10^3^ mV^2^, respectively). The additional 280 nm absorbance peak, corresponding to the column void volume, observed in the elution profile of *Bc*GR incubated with compound **(2)** is presumably related only to the compound. This peak is not present in the fluorescence traces (S7 Fig), and no protein could be detected in corresponding fractions when loaded on SDS-PAGE (S8 Fig). Therefore, the content of this peak was not considered in further analyses.

In presence of both substrate (D-Glu) and either inhibitor **(1)** or **(2)**, an additional elution peak corresponding to molecular species of approximately 70 kDa appeared in the chromatograms, suggesting enzyme dimerization (Fig 4). On the contrary, when *Bc*GR was incubated with inhibitors but in the absence of substrate, such peak was absent and the enzyme was entirely in monomeric form; the same was observed in the presence of D-Glu only, as already shown in Fig 2. To ensure that the dimerization observed was specific to *Bc*GR, the two unrelated proteins BSA and lysozyme were used as controls. In all conditions tested, the two protein eluted as monomers, thus excluding unspecific cross-linking effects of the compounds (S6 Fig).

**Fig 5:**
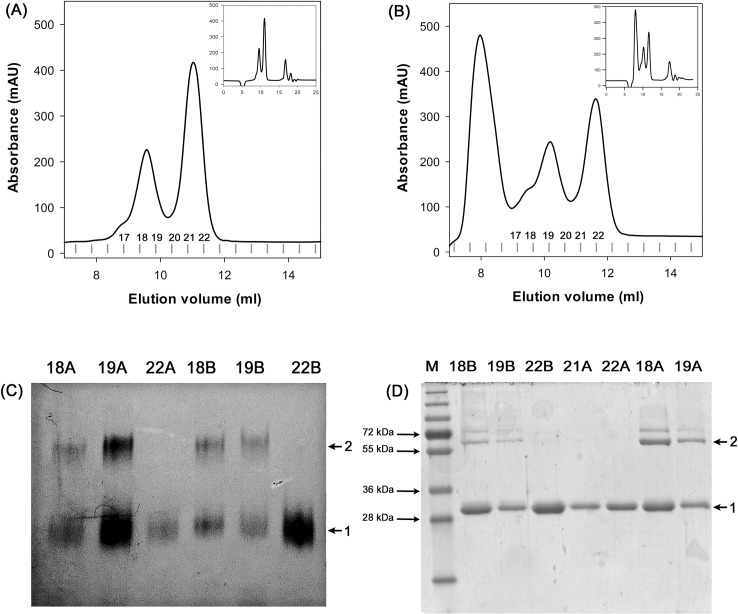
Influence of compounds on the oligomeric state of *Bc*GR. Determination of the oligomerization state of *Bc*GR in the presence of substrate and **(1)** or **(2)** complexes was confirmed by BN gel electrophoresis and chemical cross-linking analyses. In both cases protein samples (84 μM, in a final volume of 500 μl) were incubated with compounds in the ratio of molar concentration of protein (1:4) and in the presence of 30 mM D-Glu and loaded on a Superdex 75 10/300 GL column (Panels A and B show a magnification of the run, represented in the insets). Fractions corresponding to absorbance peaks were then analyzed by BN gel electrophoresis (C) and chemical cross-linking (D). The arrows indicate monomeric (1) and dimeric (2) oligomeric species.

To confirm the dimerization of *Bc*GR, additional gel filtration analyses were performed using a semi-preparative Superdex 75 10/300 GL column; in this case, the eluted fractions were collected and analyzed by both BN gel electrophoresis and chemical cross-linking (Fig 5). The presence of a dimeric form of the protein was confirmed in the 70 kDa peak, whilst the protein eluting in the 30 kDa peak was exclusively monomeric (Fig 5C and 5D).

It is noteworthy that, although the compounds were in molar excess (from 3 to 5-fold) with respect to the total protein concentration, the *Bc*GR was always found only partially in the dimeric form. In addition, when the sample eluted in the 70 kDa peak was reloaded, again it eluted in two separate peaks, corresponding to monomeric and dimeric forms of the protein. The integration of the peak area revealed a 2:1 monomer-dimer ratio for compound (1) (peak areas 319 mV^2^ and 159 mV^2^, respectively) and of 1.5:1 for compound (2) (peak areas 202 mV^2^ and 138 mV^2^), thus indicating a labile equilibrium between the two oligomeric states induced by the compound (S9 Fig).

### 1,3,5-triazapentadienate metal complexes affect *B*. *cenocepacia* growth at very high concentrations

8 to 1024 μg/ml of the Zn (II) and Mn (III) 1,3,5-triazapentadienate **(1)** and **(2)**, were added to planktonic *B*. *cenocepacia* J2315 cells. Bacterial cells were able to grow in the presence of up to 256 μg/ml of both compounds, demonstrating that at least 512 μg/ml of the compounds are necessary to inhibit their growth.

## Discussion

The resistance of *Burkholderia cepacia* complex (Bcc) to an extensive range of antibiotic classes has prompted for the search of novel antibacterial agents and novel targets [2]. Being involved in the formation of D-glutamate, an essential component of the peptidoglycan, glutamate racemase is recognized as a valuable target for identification and design of new candidate drugs [53]. In this context, we produced and characterized the *B*. *cenocepacia* J2315 glutamate racemase (*Bc*GR), to perform compound screening with the aim to identify new potential inhibitors.

Using recombinant *Bc*GR produced in *E*. *coli*, we biochemically characterized its ability to convert D-Glu into L-Glu, and found that the kinetic parameters fall within the same range of the other characterized bacterial GRs [29, 42].

Being the recombinant enzyme useful for inhibitor screening, we initially tested a series of compounds already known to be inhibitors of GRs from different sources, such as 1H-benzimidazole-2-sulfonic acid, dipicolinic acid (DPA), 4-hydroxybenzene-1,3-disulfonate, and (2R)-2-amino-4-benzylpentanedioic acid. Among these compounds, we found that only DPA was able to inhibit *Bc*GR, with an IC_50_ value of 0.15 ± 0.03 mM. Interestingly, DPA proved to be a non-competitive inhibitor of GR from *B*. *anthracis* with a similar efficacy (K_i_ = 92 μM) [25], whilst it was only a very weak inhibitor of other GR (i.e. GR from *B*. *subtilis*, K_i_ = 2.7 mM) [23]. On the contrary, 1-H-benzimidazole-2-sulfonic acid and 4-hydroxybenzene-1,3-disulfonate, which are considered potent inhibitors of GR from *B*. *subtilis* (K_i_ = 9 μM) [45] and *B*. *anthracis* (K_i_ = 59 μM) [26], did not show any inhibition towards *Bc*GR, as well as (2R)-2-amino-4-benzylpentanedioic. Indeed the latter compound was demonstrated to be ineffective against GR enzymes bearing a Val residue at position 149 of the active site (157 V for *Bc*GR enzyme) [45], as *Bc*GR does.

Afterwards, we screened an in-house chemical library of compounds with antimicrobial activity. This screening allowed us to identify two hit compounds, the bis(2,4-bis(trichloromethyl)-1,3,5-triazapentadienato)-zinc(II) complex **(1)** and the tris(2,4-bis(trichloromethyl)-1,3,5-triazapentadienate)-Mn(III) complex **(2)**, which inhibited *Bc*GR almost completely. The two compounds were found to be effective non-competitive inhibitors, showing IC_50_ values in the micromolar range. In order to assess whether enzyme inhibition was possibly induced by the metal ions present in the complexes, we determined the effect of free Zn^2+^ and Mn^2+^ on the activity of *Bc*GR, finding that effectively both affected *Bc*GR activity (S5 Fig). However, in the presence of an excess of EDTA the inhibition by divalent ions completely disappeared, whilst the inhibitory effect of **(1)** and **(2)** could be observed also in the presence of EDTA, thus proving a direct effect for the metal-containing compounds. Unfortunately, the two compounds show an effect against *B*. *cenocepacia* viability only at very high concentrations, indicating that probably, due to their high molecular weight (674.94 and 1047.41, respectively), they cannot efficiently penetrate the bacterial cells.

Since most GRs have been reported to exist in equilibrium between monomeric and dimeric states depending on the presence or absence of substrates or ligands [21, 42–44], we evaluated *Bc*GR oligomerization and determined the effects of the **(1)** and **(2)** complexes on the oligomeric state. Native *Bc*GR showed to be a monomer both in the presence and absence of substrates (L- or D-glutamate), as well as in the presence of inhibitory compounds. Analysis of the surface charge distribution in the *Bc*GR homology model supports the observed monomeric nature of the enzyme, as *Bc*GR seems to lack several critical complementary electrostatic regions that enable dimerization in homologous enzymes (S2 Fig). On the contrary, by mixing the enzyme with both substrate and inhibitors **(1)** or **(2)**, we consistently detected *Bc*GR dimerization. The formation of a protein dimer in the presence of the compounds has been observed specifically for *Bc*GR, and only in the presence of the substrate. Together, this experimental observation and the non-competitive nature of the inhibition, suggest that the compounds may bind to the enzyme-substrate complex, promoting the formation of a non-productive oligomeric state of the enzyme. In this respect, it is noteworthy that previous molecular dynamics and site directed mutagenesis studies on the *E*. *coli* RacE significantly indicated that the monomeric form might be more active than the dimeric [54], further supporting our identification of inhibitor-induced non-productive *Bc*GR oligomers. Nevertheless, high resolution structural data on the dimeric *Bc*GR:substrate:inhibitor triple complexes and their comparison with the enzyme in monomeric form would greatly help to understand the mechanism of action of this new class of inhibitor compounds.

In conclusion, our characterization of recombinant *Bc*GR may represent a promising starting point for screening and/or drug design for the development of novel inhibitors effective against *B*. *cenocepacia*. The newly identified metal ion-containing compounds inhibit *Bc*GR by modulating the oligomerization state of enzyme:substrate:inhibitor triple complexes, offering an attractive novel strategy to target *Bc*GR enzyme function, possibly overcoming some of the difficulties associated to the treatment of bacterial infections [55].

## Supporting Information

S1 Fig Homology model of *Bc*GR. (A) Sequence alignment of *Bc*GR with homologous GR with known three-dimensional structures. Colors highlight the three-dimensional organization of the enzyme family, with the two major domains shown in orange and blue and the C-terminal helix in red. (B) Cartoon representation of the *Bc*GR homology model. Colors as in (A). The location of the substrate binding pocket inferred by superposition analysis with homologous GR structures is shown with a D-glutamate molecule depicted as green ball-and sticks.
(PDF)

S2 Fig Comparison of dimerization interfaces observed in three-dimensional structures of GR enzymes. On the left, the six different oligomerization states identified by PISA are shown. For clarity, one monomer (magenta) is always shown in the same orientation, and substrate molecules identified in the various structures (either D-Glu or D-Gln) are shown with yellow spheres. On the right, the surface charge distribution (colored from -5 k_b_Te_c_^-1^ (red) to +5 k_b_Te_c_^-1^ (blue)) of the dimerization interface is shown for the *Bc*GR homology model and for representative GRs displaying that specific type of dimerization in the crystal structures. The black outline shows the position of the second molecule generating the dimer on the contact interface. For *Bc*GR, the putative dimeric interface has been generated by superposing two copies *Bc*GR homology models on the two monomers of a dimeric structure identified with specific dimerization interface type.
(PDF)

S3 Fig Inhibition of *Bc*GR activity by dipicolinic acid. IC_50_ value was determined at 20 mM of D-Glu, by fitting the experimental data as reported in Materials and Methods.
(PDF)

S4 Fig **Kinetic analysis of *Bc*GR in the presence of different concentrations of compound (1) (panel A) or compound (2) (panel B).** *Bc*GR enzyme activity was determined at four different concentrations of D-Glu (range 5–50 mM), in the presence of five different concentrations of compounds (range 0–100 μM). The Lineweaver-Burk plot of the data reveals the non-competitive nature of the inhibition.
(PDF)

S5 Fig Inhibition of *Bc*GR activity by ZnCl_2_ and MnCl_2_. IC_50_ values of ZnCl_2_ (●) and MnCl_2_ (▼) were determined at 20 mM of D-Glu, by fitting the experimental data as reported in Materials and Methods.
(PDF)

S6 Fig Superdex 75 5/150 GL elution profiles of the control proteins in the presence of (1) and (2) compounds. Tryptophan fluorescence traces of bovine serum albumin (panel A) and chicken egg lysozyme (panel B), after incubation with 0.1 mM compound **(1)** or **(2)**, in the absence or in the presence of D-Glu (10 mM). From bottom to top: no addition; with 0.1 mM **(1)**; with 0.1 mM **(1)** and 10 mM D-Glu; with 0.1 mM **(2)**; with 0.1 mM **(2)** and 10 mM D-Glu. Figure is representative of two independent experiments.
(PDF)

S7 Fig Absorbance and fluorescence chromatographic profile on Superdex 75 5/150 GL column of *Bc*GR incubated with compound (2). (PDF)

S8 Fig SDS-PAGE (A) of the exclusion volume fraction peak of the gel filtration of *Bc*GR incubated with compound (2), revealing that the absorbance detected is not related to the protein. (PDF)

S9 Fig Reload of *Bc*GR on gel filtration column. The protein eluted from the 70 kDa peak in the presence of substrate compound (1) and compound (2) were pooled and loaded again on the column. Protein eluted again in two peaks, thus indicating an equilibrium between monomeric and dimeric form. Fractions were analysed by chemical cross-linking (C). The oligomeric species are indicated by the arrows.
(PDF)

S1 Table List of *Bc*GR structural homologs and analysis of their oligomeric state. (PDF)
