# Supplementary material for: Biochemical Characterization of Glutamate Racemase—A New Candidate Drug Target against Burkholderia cenocepacia Infections
Source: PLoS One. 2016 Nov 29;11(11):e0167350. doi: 10.1371/journal.pone.0167350 (PMC5127577; doi:10.1371/journal.pone.0167350)
Supplement: S2 Fig — On the left, the six different oligomerization states identified by PISA are shown. For clarity, one monomer (magenta) is always shown in the same orientation, and substrate molecules identified in the various structures (either D-Glu or D-Gln) are shown with yellow spheres. On the right, the surface charge distribution (colored from -5 kbTec-1 (red) to +5 kbTec-1 (blue)) of the dimerization interface is shown for the BcGR homology model and for representative GRs displaying that specific type of dimerization in the crystal structures. The black outline shows the position of the second molecule generating the dimer on the contact interface. For BcGR, the putative dimeric interface has been generated by superposing two copies BcGR homology models on the two monomers of a dimeric structure identified with specific dimerization interface type. (PDF) [file pone.0167350.s002.pdf]

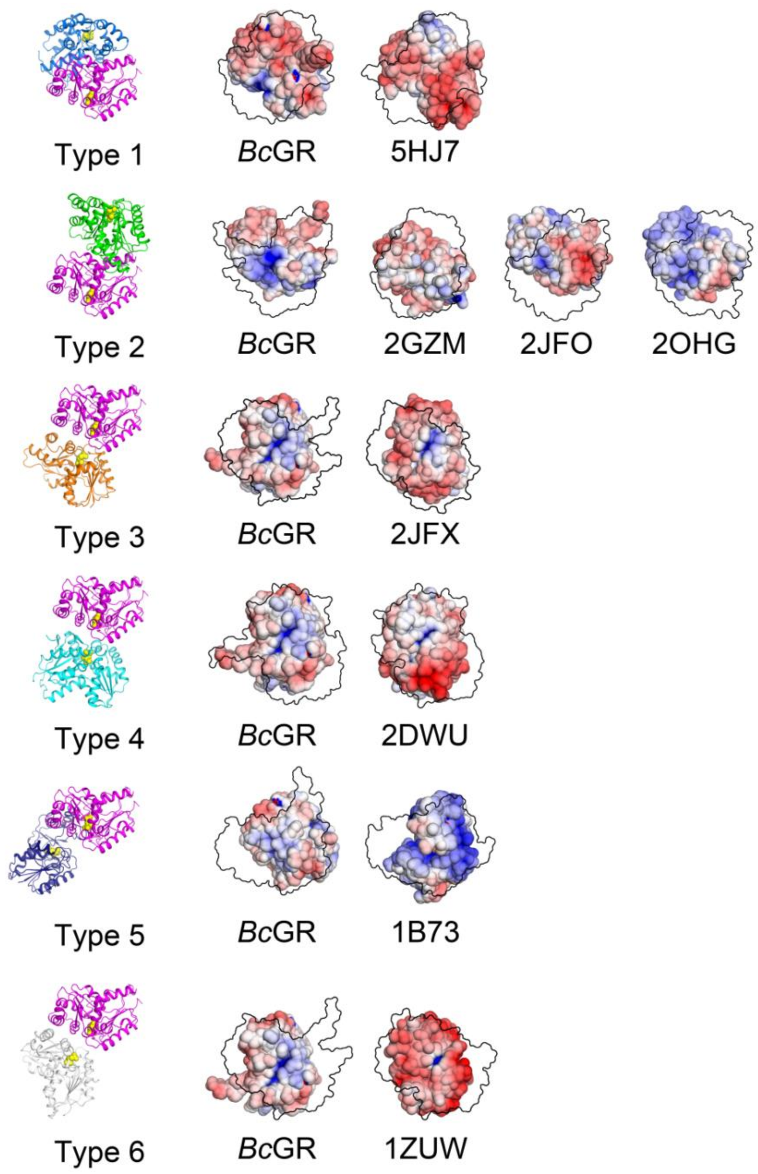

S2 Fig. Comparison of dimerization interfaces observed in three-dimensional structures of GR enzymes. On the left, the six different oligomerization states identified by PISA are shown. For clarity, one monomer (magenta) is always shown in the same orientation, and substrate molecules identified in the various structures (either D-Glu or D-Gln) are shown with yellow spheres. On the right, the surface charge distribution (colored from -5 $k_bTe_c^{-1}$ (red) to +5 $k_bTe_c^{-1}$ (blue)) of the dimerization interface is shown for the *Bc*GR homology model and for representative GRs displaying that specific type of dimerization in the crystal structures. The black outline shows the position of the second molecule generating the dimer on the contact interface. For *Bc*GR, the putative dimeric interface has been generated by superposing two copies *Bc*GR homology models on the two monomers of a dimeric structure identified with specific dimerization interface type.
