# Supplementary material for: Biochemical Characterization of Glutamate Racemase—A New Candidate Drug Target against Burkholderia cenocepacia Infections
Source: PLoS One. 2016 Nov 29;11(11):e0167350. doi: 10.1371/journal.pone.0167350 (PMC5127577; doi:10.1371/journal.pone.0167350)
Supplement: S4 Fig — Kinetic analysis of BcGR in the presence of different concentrations of compound (1) (panel A) or compound (2) (panel B). BcGR enzyme activity was determined at four different concentrations of D-Glu (range 5–50 mM), in the presence of five different concentrations of compounds (range 0–100 μM). The Lineweaver-Burk plot of the data reveals the non-competitive nature of the inhibition. (PDF) [file pone.0167350.s004.pdf]

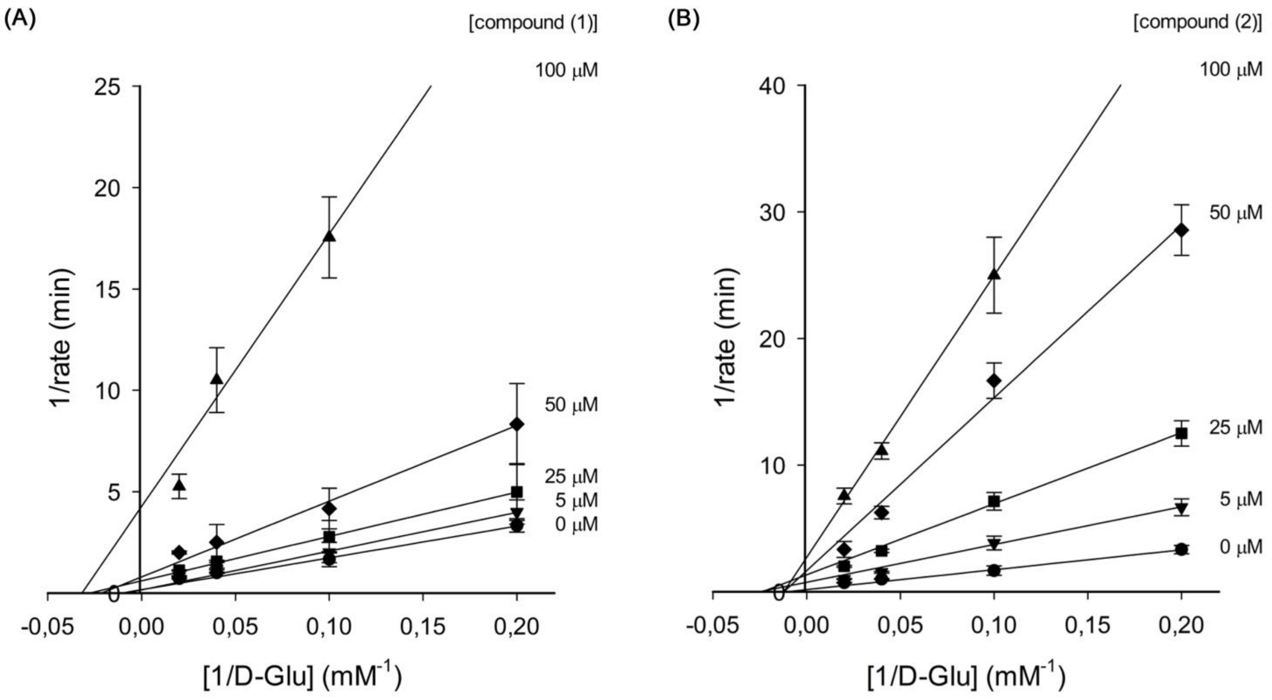

S4 Fig. Kinetic analysis of *Bc*GR in the presence of different concentrations of compound (1) (panel A) or compound (2) (panel B). *Bc*GR enzyme activity was determined at four different concentrations of D-Glu (range 5-50 mM), in the presence of five different concentrations of compounds (range 0-100 µM). The Lineweaver-Burk plot of the data reveals the non-competitive nature of the inhibition.
