# Supplementary material for: Biochemical Characterization of Glutamate Racemase—A New Candidate Drug Target against Burkholderia cenocepacia Infections
Source: PLoS One. 2016 Nov 29;11(11):e0167350. doi: 10.1371/journal.pone.0167350 (PMC5127577; doi:10.1371/journal.pone.0167350)
Supplement: S5 Fig — IC50 values of ZnCl2 (●) and MnCl2 (▼) were determined at 20 mM of D-Glu, by fitting the experimental data as reported in Materials and Methods. (PDF) [file pone.0167350.s005.pdf]

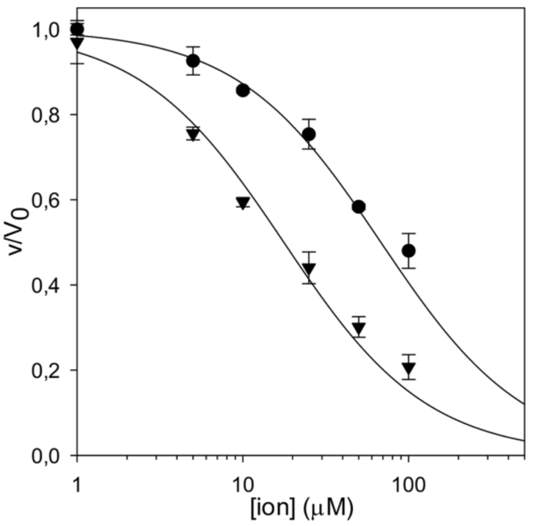

S5 Fig. Inhibition of *Bc*GR activity by $ZnCl_2$ and $MnCl_2$. IC_{50} values of $ZnCl_2$ (●) and $MnCl_2$ (▼) were determined at 20 mM of D-Glu, by fitting the experimental data as reported in Materials and Methods.
