# Supplementary material for: Biochemical Characterization of Glutamate Racemase—A New Candidate Drug Target against Burkholderia cenocepacia Infections
Source: PLoS One. 2016 Nov 29;11(11):e0167350. doi: 10.1371/journal.pone.0167350 (PMC5127577; doi:10.1371/journal.pone.0167350)
Supplement: S6 Fig — Tryptophan fluorescence traces of bovine serum albumin (panel A) and chicken egg lysozyme (panel B), after incubation with 0.1 mM compound (1) or (2), in the absence or in the presence of D-Glu (10 mM). From bottom to top: no addition; with 0.1 mM (1); with 0.1 mM (1) and 10 mM D-Glu; with 0.1 mM (2); with 0.1 mM (2) and 10 mM D-Glu. Figure is representative of two independent experiments. (PDF) [file pone.0167350.s006.pdf]

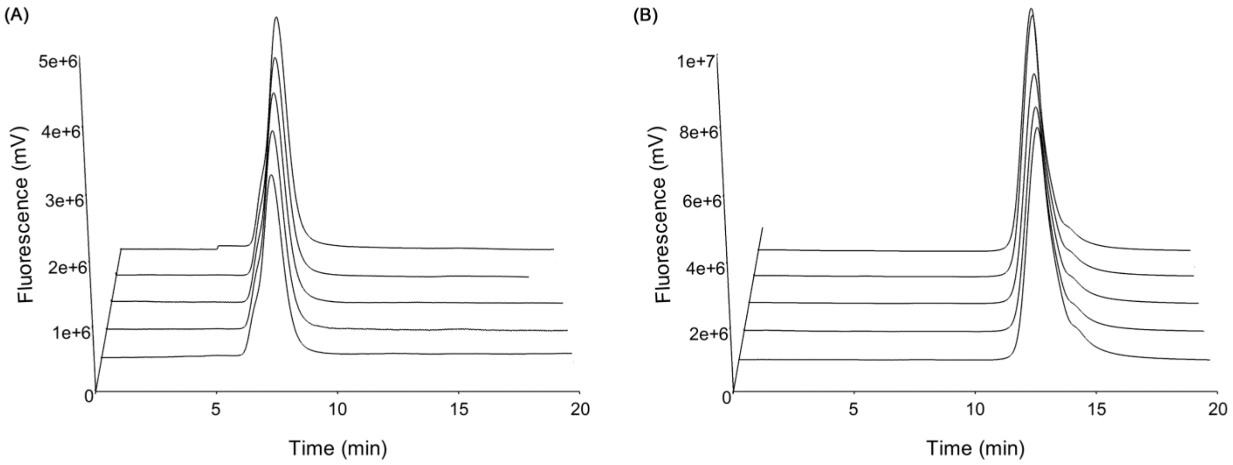

S6 Fig. Superdex 75 5/150 GL elution profiles of the control proteins in the presence of (1) and (2) compounds. Tryptophan fluorescence traces of bovine serum albumine (panel A) and chicken egg lysozyme (panel B), after incubation with 0.1 mM compound **(1)** or **(2)**, in the absence or in the presence of D-Glu (10 mM). From bottom to top: no addition; with 0.1 mM **(1)**; with 0.1 mM **(1)** and 10 mM D-Glu; with 0.1 mM **(2)**; with 0.1 mM **(2)** and 10 mM D-Glu. Figure is representative of two independent experiments.
