# Supplementary material for: Biochemical Characterization of Glutamate Racemase—A New Candidate Drug Target against Burkholderia cenocepacia Infections
Source: PLoS One. 2016 Nov 29;11(11):e0167350. doi: 10.1371/journal.pone.0167350 (PMC5127577; doi:10.1371/journal.pone.0167350)
Supplement: S8 Fig — (PDF) [file pone.0167350.s008.pdf]

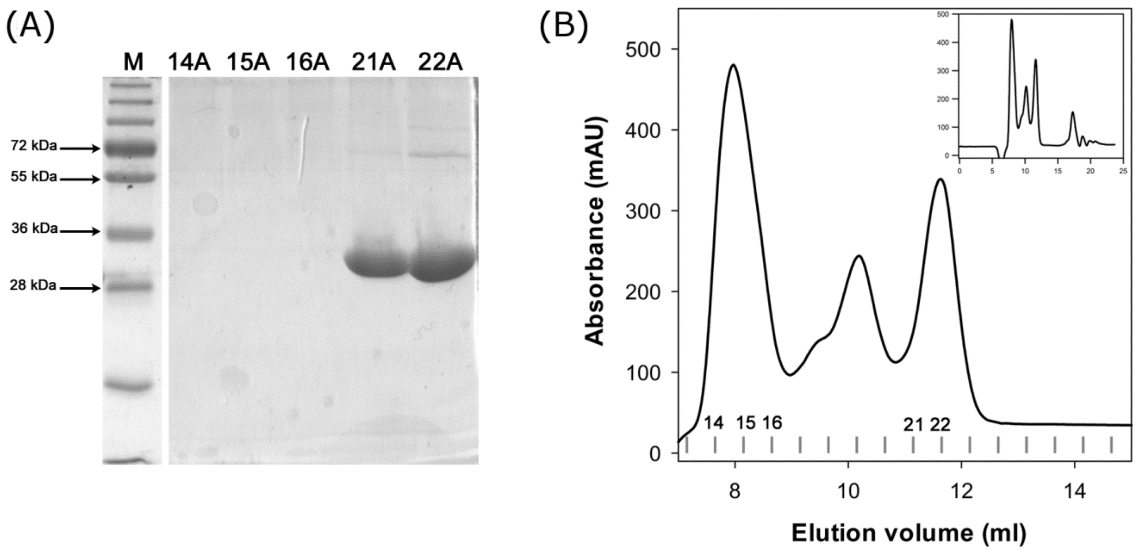

S8 Fig. SDS-PAGE (A) of the exclusion volume fraction peak of the gel filtration of *Bc*GR incubated with compound (2), revealing that the absorbance detected is not related to the protein.
