# Supplementary material for: Biochemical Characterization of Glutamate Racemase—A New Candidate Drug Target against Burkholderia cenocepacia Infections
Source: PLoS One. 2016 Nov 29;11(11):e0167350. doi: 10.1371/journal.pone.0167350 (PMC5127577; doi:10.1371/journal.pone.0167350)
Supplement: S9 Fig — The protein eluted from the 70 kDa peak in the presence of substrate compound (1) and compound (2) were pooled and loaded again on the column. Protein eluted again in two peaks, thus indicating an equilibrium between monomeric and dimeric form. Fractions were analysed by chemical cross-linking (C). The oligomeric species are indicated by the arrows. (PDF) [file pone.0167350.s009.pdf]

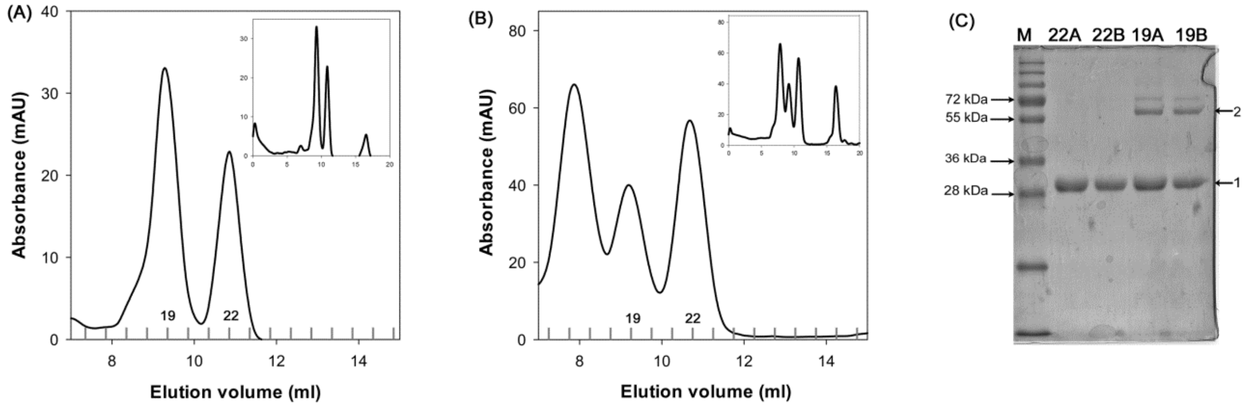


**S9 Fig. Reload of *Bc*GR on gel filtration column.** The protein eluted from the 70 kDa peak in the presence of substrate compound (1) and compound (2) were pooled and loaded again on the column. Protein eluted again in two peaks, thus indicating an equilibrium between monomeric and dimeric form. Fractions were analysed by chemical cross-linking (C). The oligomeric species are indicated by the arrows.
