# Supplementary material for: Biochemical Characterization of Glutamate Racemase—A New Candidate Drug Target against Burkholderia cenocepacia Infections
Source: PLoS One. 2016 Nov 29;11(11):e0167350. doi: 10.1371/journal.pone.0167350 (PMC5127577; doi:10.1371/journal.pone.0167350)
Supplement: S1 Fig — (A) Sequence alignment of BcGR with homologous GR with known three-dimensional structures. Colors highlight the three-dimensional organization of the enzyme family, with the two major domains shown in orange and blue and the C-terminal helix in red. (B) Cartoon representation of the BcGR homology model. Colors as in (A). The location of the substrate binding pocket inferred by superposition analysis with homologous GR structures is shown with a D-glutamate molecule depicted as green ball-and sticks. (PDF) [file pone.0167350.s001.pdf]

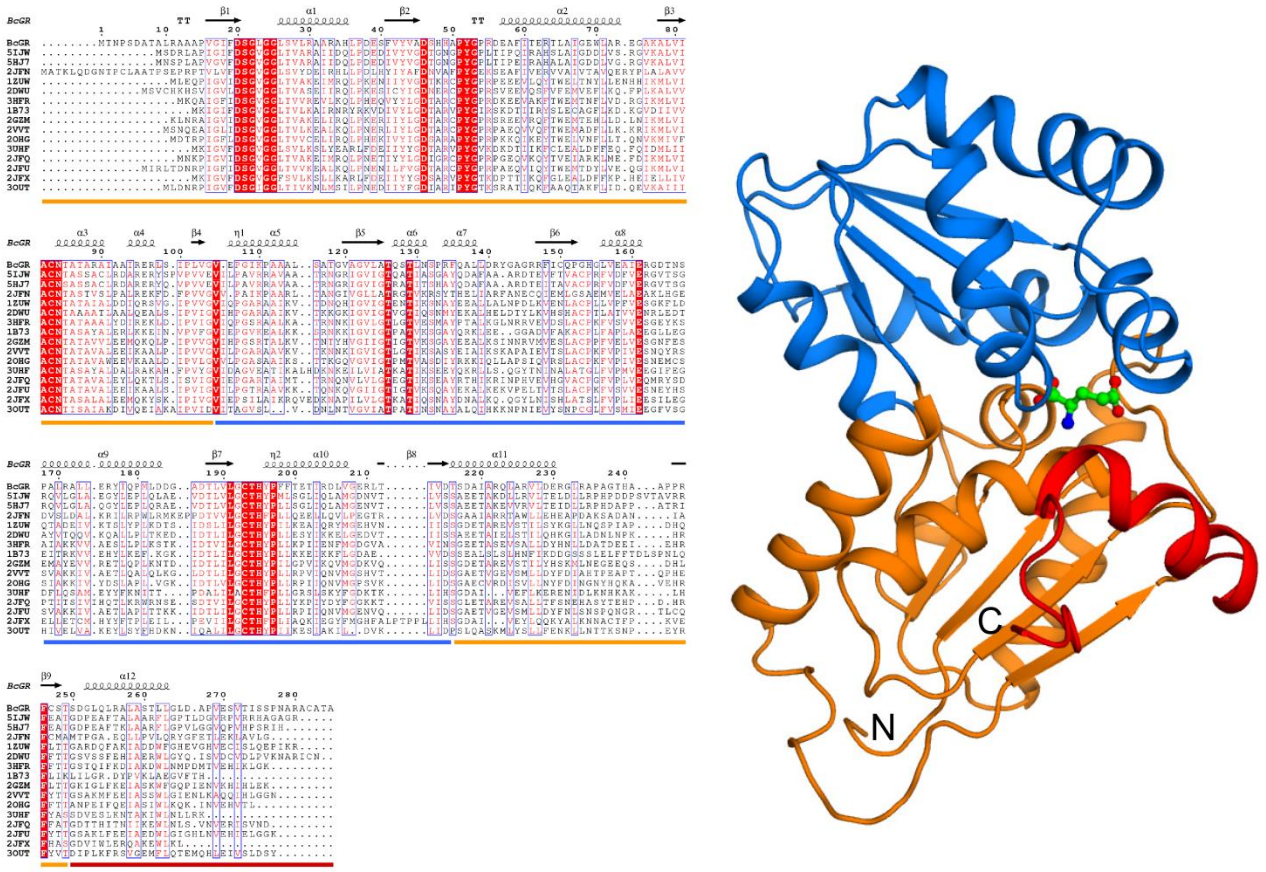

S1 Fig. Homology model of *Bc*GR. (A) Sequence alignment of *Bc*GR with homologous GR with known three-dimensional structures. Colors highlight the three-dimensional organization of the enzyme family, with the two major domains shown in orange and blue and the C-terminal helix in red. (B) Cartoon representation of the *Bc*GR homology model. Colors as in (A). The location of the substrate binding pocket inferred by superposition analysis with homologous GR structures is shown with a D-glutamate molecule depicted as green ball-and sticks.
