# Supplementary material for: Biochemical Characterization of Glutamate Racemase—A New Candidate Drug Target against Burkholderia cenocepacia Infections
Source: PLoS One. 2016 Nov 29;11(11):e0167350. doi: 10.1371/journal.pone.0167350 (PMC5127577; doi:10.1371/journal.pone.0167350)
Supplement: S1 Table — (PDF) [file pone.0167350.s010.pdf]

S1 Table. List of *Bc*GR structural homologs and analysis of their oligomeric state

Protein	PDB ID	Sequence Identity (%)	Oligomeric state[1]	Reference
MurI from *M. smegmatis*	5JWV	41	Dimer, type 1	N/A[2]
MurI from *M. tuberculosis*	5HJ7	40	Dimer, type 1	N/A[2]
MurI from *E. coli*	2JFN	38	Monomer	[13]
RacE from *B. subtilis*	1ZUW	32%	Dimer, type 2A<br>Dimer, type 6	[16]
RacE1 from *B. anthracis*	2DWU	32%	Dimer, type 2A<br>Dimer, type 4	[31]
MurI from *L. monocytogenes*	3HFR	32%	Dimer, type 2B	N/A[2]
MurI from *A. pyrophilus*	1B73	32%	Dimer, type 5	[12]
RacE2 from *B. anthracis*	2GZM	31%	Dimer, type 2A	[31]
MurI from *E. faecalis*	2JFO, 2VVT	31%	Dimer, type 2B	[13,15]
MurI from *S. pyogenes*	2OHG, 2OHO	31%	Dimer, type 2C	[17]
MurI from *C. jejuni*	3UHF	31%	Dimer, type 3	N/A[2]
MurI from *S. aureus*	2JFQ	30%	Dimer, type 2A	[13]
MurI from *E. faecium*	2JFU	30%	Dimer, type 2C	[13]
MurI from *H. pylori*	2JFX	30%	Dimer, type 3	[13]
MurI from *F. tularensis*	3OUT	29%	Dimer, type 3	N/A[2]

[1] Evaluation of oligomeric state based on PISA analysis (Krissinel and Henrick. Inference of macromolecular assemblies from crystalline state. 2007 J Mol Biol. 372, 774-797) of the three-dimensional structures available on the PDB. Dimer types are described in supplementary figure Fig. S2.

[2] there is no publication associated to this entry in the protein data bank.
